# Mitochondrial Genome Contributes to the Thermal Adaptation of the Oomycete *Phytophthora infestans*

**DOI:** 10.3389/fmicb.2022.928464

**Published:** 2022-06-28

**Authors:** Lin-Lin Shen, Abdul Waheed, Yan-Ping Wang, Oswald Nkurikiyimfura, Zong-Hua Wang, Li-Na Yang, Jiasui Zhan

**Affiliations:** ^1^Institute of Oceanography, Minjiang University, Fuzhou, China; ^2^Sichuan Provincial Key Laboratory for Development and Utilization of Characteristic Horticultural Biological Resources, Chengdu Normal University, Chengdu, China; ^3^Institute of Plant Virology, Fujian Agriculture and Forestry University, Fuzhou, China; ^4^Department of Forest Mycology and Plant Pathology, Swedish University of Agricultural Sciences, Uppsala, Sweden

**Keywords:** mitochondria, evolutionary ecology, population genetic, local adaptation, agricultural pathogen, climate change

## Abstract

As a vital element of climate change, elevated temperatures resulting from global warming present new challenges to natural and agricultural sustainability, such as ecological disease management. Mitochondria regulate the energy production of cells in responding to environmental fluctuation, but studying their contribution to the thermal adaptation of species is limited. This knowledge is needed to predict future disease epidemiology for ecology conservation and food security. Spatial distributions of the mitochondrial genome (mtDNA) in 405 *Phytophthora infestans* isolates originating from 15 locations were characterized. The contribution of MtDNA to thermal adaptation was evaluated by comparative analysis of mtDNA frequency and intrinsic growth rate, relative population differentiation in nuclear and mtDNA, and associations of mtDNA distribution with local geography climate conditions. Significant variation in frequency, intrinsic growth rate, and spatial distribution was detected in mtDNA. Population differentiation in mtDNA was significantly higher than that in the nuclear genome, and spatial distribution of mtDNA was strongly associated with local climatic conditions and geographic parameters, particularly air temperature, suggesting natural selection caused by a local temperature is the main driver of the adaptation. Dominant mtDNA grew faster than the less frequent mtDNA. Our results provide useful insights into the evolution of pathogens under global warming. Given its important role in biological functions and adaptation to local air temperature, mtDNA intervention has become an increasing necessity for future disease management. To secure ecological integrity and food production under global warming, a synergistic study on the interactive effect of changing temperature on various components of biological and ecological functions of mitochondria in an evolutionary frame is urgently needed.

## Introduction

Local adaptation is a phenomenon whereby a member of species demonstrate a higher fitness, here defined as the competitive ability of survival, development, reproduction, and transmission (Kaltz and Shykoff, 1998; Giraud et al., 2017), in its native environment than other members of the same species that domicile elsewhere (Hoeksema and Forde, 2008). Phenotypic and genetic differentiation of species along defined ecological gradients, such as a continuous variation in thermal niches and altitudes (Roy et al., 2015), can also be used to infer local adaptation. This pattern of adaptation results from a complex interaction among members of a species as well as between species and the environment or is regulated by eco-evolutionary processes involving in genetic and phenotypic modification of genome structure and expression (Consuegra et al., 2015). Although local adaptation has been a paradigm of evolutionary study (Giraud et al., 2017), knowledge related to the topic is still fragmented. For example, local adaptation involves trades-offs and synergies among various ecological traits in a species, and it is unclear how different parts of a genome (e.g., nuclear vs. organelle) function together to shape the adaptation.

Natural selection and migration are two key evolutionary processes determining the pattern of species' adaptation to ecological gradients due to their counter-effect on the accumulation of the genetic variation required for phenotypic clines (Santangelo et al., 2018). Depending on their relative contribution to population genetic structure, interactions between these processes may increase, retain, or even decrease the performance of species members in their native ecosystems (Kaltz and Shykoff, 1998), leading to local adaptation, no adaptation, and mal-adaptation, respectively. Other evolutionary processes regulating genetic variation also contribute to the pattern and rate of local adaptation. For example, population extinctions or re-colonization associated with frequent bottlenecks or founder effects can prevent the development of local adaptation (Weaver et al., 2021). Phenotypic plasticity generated by gene expression and biochemical network regulation can also hamper local adaptation.

As an important component of ecosystems, temperature is expected to play an important role in local adaptation. By regulating genomic resilience and cellular activity, such as replication of proofreading (Barshis et al., 2013), transcription and translation profiles (Morgan et al., 2018), enzyme stability (Arcus et al., 2020), respiration, signal transduction (Xiang et al., 2021) etc., temperature exerts a critical influence on genetic, biological, ecological, and evolutionary processes of species such as mutation rate, survival, reproduction, competition, and dispersal. For example, an increased extinction rate associated with elevated temperatures was documented from a 30-year interaction between *Triphragmium ulmariae* and *Filipendula ulmaria* in Sweden (Zhan et al., 2018). An elevation of selection intensity and mutation rate that resulted from increasing temperature was observed in a genome-wide analysis of seed beetle (Berger et al., 2021). Adaptive evolution mediated by local temperature was also documented at an organismal level in many other species (Zhan and McDonald, 2011; Roy et al., 2015; He et al., 2018), including plant pathogens *Zymoseptoria tritici* (Zhan and McDonald, 2011) and *Rhynchosporium commune* (Stefansson et al., 2013).

Mitochondria are the organelles in which chemical energy required for cellular processes is generated (Roger et al., 2017). They can be found in the cytoplasm of eukaryotic organisms. The organelles have their own genome that is independent of the nucleus, but substantially like prokaryotic bacteria (Roger et al., 2017). Mitochondrial genomes (MtDNA) usually demonstrate a faster evolutionary rate than their nuclear counterparts, particularly in many species of mammal and plant species, and exhibit uniparental inheritance or reduced recombination (Popadin et al., 2012) even for species involving a biparental inheritance. These genetic and biological characteristics make mtDNA a ubiquitous material to address many evolutionary questions (Zhan et al., 2004), such as experimental tests for species adaptation. The feature of fast evolution enables the detection of genomic changes in a reasonable time (Zhan and McDonald, 2011; Yang et al., 2016). With uniparental inheritance, allelic polymorphisms are exclusively generated by mutation, removing potential genetic noise caused by recombination events (Popadin et al., 2012).

In addition to supplying energy for cellular activities, mtDNA is involved in many other biological and biochemical processes, such as calcium storage (Deline et al., 2021), cellular proliferation, signal conduction, etc. Interestingly, mtDNA has long been assumed to be exempted from natural selection (Galtier et al., 2009) and used to address evolutionary questions that require neutral markers, such as phylogenetic analysis of species relationships (Mao et al., 2020). Experimental evaluation of mitochondrial contribution to ecological adaptation such as thermal adaptation is limited, especially in plant pathogens. Therefore, the overall aim of the current study is to investigate the role of mtDNA in species adaptation to thermal conditions using *P. infestans*, a pathogenic microbe that originated from potato plants in agricultural ecosystems.

*Phytophthora infestans* is an infamous plant pathogen, which can greatly reduce the fitness of potatoes and tomatoes by damaging leaves, stems, and other parts of host tissues (Leesutthiphonchai et al., 2018). The pathogen has a worldwide distribution but thrives best under high humidity and temperate conditions (Leesutthiphonchai et al., 2018). Although it is mainly reproduced asexually, *P. infestans* is observed with a high capacity (Li et al., 2012) for acquiring resistance to host immunity (Yang et al., 2020) and tolerance to fungicide (Chen et al., 2018) and environmental stresses genetically or physiologically (Wu et al., 2019, 2020; Lurwanu et al., 2020; Wang et al., 2020), possibly attributed to its large genome enriched with transposable elements (Haas et al., 2009), short epidemic cycle (Leesutthiphonchai et al., 2018), and continuous gene flow (Dey et al., 2018). Indeed, *P. infestans* can complete its reproduction cycles and destroy entire plants in a few days when environmental conditions are conducive (Harrison, 1992). It has rendered the effectiveness of naïve host resistance within a few years of being encountered (Forbes, 2012) and quickly increased fitness after a short period of training under thermal stresses (Wu et al., 2020).

*The* mtDNA *of P. infestans* is ~40 kb in size with 53 predicted protein-coding genes (Avila-Adame et al., 2006; Patil et al., 2017), much smaller compared to its nuclear counterpart. In addition to the common features of uniparental inheritance (probably maternal inheritance), rapid mutation, and non-recombination (Avila-Adame et al., 2006), *P. infestans* mtDNA is easy to work with and has shown spatial heterogeneity of genetic polymorphism (Yang et al., 2013), making it a useful material to study the thermal adaptation of species.

To reach the overarching aim, we molecularly assayed the mitochondrial profile of more than 400 *P. infestans* isolates originating from geographic locations along environmental gradients across a major potato production country in the world. The spatial population genetic structure of the mitochondrial genome was contrasted to climatic conditions (temperature, rainfall, and solar radiation) in the collection sites. Phenotypic values of mitochondrial types were quantified using intrinsic growth rate and linked to their competitive ability. The specific objectives of the research were to: (1) understand the spatial distribution of mitochondrial haplotypes in *P. infestans;* (2) reveal the relative importance of genetic drift and natural selection on the spatial distribution of mtDNA; (3) evaluate how meteorological factors and geographic positions affect the spatial distribution of *P. infestans* haplotypes; and (4) explore the link between the intrinsic growth rate of *P. infestans* mtDNA and its frequency in nature.

## Materials and Methods

### Spatial Origin of Pathogen Collections

**Figure 1:**
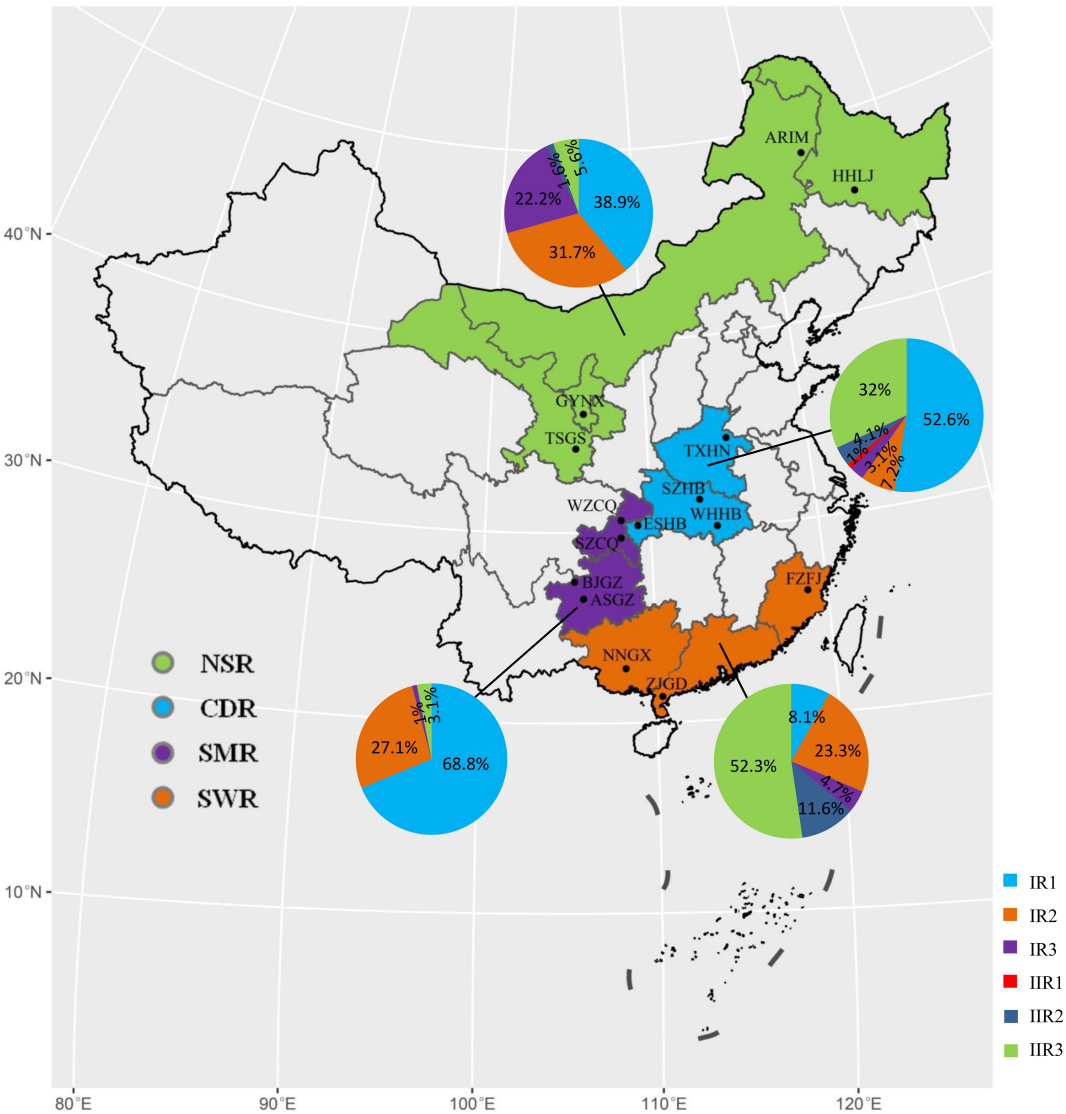
Map showing the geographic origins and frequency distribution of mitochondrial haplotypes in the *Phytophthora infestans* populations sampled from four potato cultivation areas in China.

Pathogen isolates were collected from 15 sites across the four potato production regions of China during the 2010–2013 growing seasons (Figure 1, Table 1). Among these sites, Harbin Heilongjiang (HHLJ), Arong Inner Mongolia (ARIM), Tianshui Gansu (TSGS), and Guyua Ningxia (GYNX) are located in the Northern single-cropping region (NSR); Tongxu Henan (TXHN), Enshi Hubei (ESHB), Suizhou Hubei (SZHB), and Wuhan Hubei (WHHB) are located in Central double-cropping region (CDR); Anshun Guizhou (ASGZ), Bijie Guizhou (BJGZ), Wanzhou Chongqing (WZCQ), and Shizhu Chongqing (SZCQ) are located in Southwestern multiple-cropping region (SMR); and Fuzhou Fujian (FZFJ), Zhanjiang Guangdong (ZJGD), and Nanning Guangxi (NNGX) are located in the Southern Winter-cropping region (SWR). These collection sites vary dramatically in climatic conditions due to their geographic positions (Zhu et al., 2016). SWR belongs to a tropical or subtropical monsoon climate which is characterized by hot summers and mild winters (https://www.fmprc.gov.cn/ce/cggb/eng/gyzg/jbgq/t216954.htm). NSR usually has severe cold winters and experiences little annual rainfall. However, summers in some areas can be very hot. Many areas in CDR have a long and humid summer with medium to high temperatures. Temperatures in the winters can drop well-below freezing and the airs can be wet. SMR has a mild temperature with a very wet summer, where potatoes can be grown in all seasons. In general, altitude gradually increases from East to West. Most potatoes in NSR and SMR are grown in high altitude areas while those in CDR and SWR are grown in low altitude areas.

**Table 1:** Climatic conditions and their associated geographic coordinates of the collection sites for the 15 *Phytophthora infestans* populations used in the study.

**PCA**	**Regions**	**Years**	**Sample** **size**	**AMT** **(**°**C)**	**Altitude** **(m)**	**Longitude**	**Latitude**	**AR** **(mm)**	**AID** **(h)**
NSR	HHLJ	2013	22	3.68	126	126.64°E	45.76°N	633.5	2,023.5
	ARIM	2013	36	2.75	219	123.46°E	48.13°N	758.1	2,632.1
	TSGS	2010	40	11.7	1,169	105.72°E	34.58°N	401.9	1,968.7
	GYNX	2010	28	7	1,778	106.29°E	36.00°N	458.3	2,563
CDR	TXHN	2012	21	16.17	67	114.47°E	34.48°N	467.1	1,803.7
	ESHB	2012	25	13.08	490	109.49°E	30.28°N	1,258	1,066.5
	SZHB	2012	21	16.25	71	113.37°E	31.72°N	714.9	1,558.8
	WHHB	2010	30	16.33	16	114.30°E	30.58°N	1,338	1,544
SMR	ASGZ	2011	44	14.7	1,378	105.93°E	26.25°N	851.1	1,276.4
	BJGZ	2012	24	13.08	1,490	105.29°E	27.30°N	828.5	873.2
	WZCQ	2012	14	18.08	328	108.38°E	30.81°N	1,016	1,072.9
	SZCQ	2010	14	16.75	553	108.11°E	30.00°N	1,170	1,195.4
SWR	FZFJ	2010	32	20.5	10	119.31°E	26.08°N	1,605	1,485.6
	ZJGD	2011	15	24.42	17	110.36°E	21.27°N	1,409	1,822.3
	NNGX	2011	39	22.6	79	108.32°E	22.82°N	1,253	1,663.3

*PCA, Potato cultivation areas; AMT, Annual means temperature (°C); AR, Annual rainfall (mm); AID, Annual insolation duration (h)*.

In all collections, infected leaves were sampled from potato plants separated by >100 cm. Only one infected leaf was collected from a plant and only one single-spore strain was isolated from a leaf. The isolates were genotyped by SSR assay of nuclear genomes as described (Knapova and Gisi, 2002). The details for pathogen isolation and the biological and molecular characterizations of the pathogen can be found in previous publications (Qin et al., 2016; Wu et al., 2016; Zhu et al., 2016).

### PCR Detection of Mitochondrial Haplotypes

*P. infestans* isolates retrieved from long-term storage were cultured on rye B media supplemented with 10 mg/L rifampicin and 100 mg/L ampicillin at 18°C in the dark for 2 weeks. A total of 200–300 mg of mycelia were harvested for DNA extraction using Easy Pure Plant Genomic DNA Kit (Transgen, Beijing). The genomic DNA was amplified with the two sets of primers specified to the mitochondria of *P. infestans* (Yang et al., 2013). One set of the primers (i.e., Insertion-F and Insertion-R) amplifies the HVRi region of the mtDNA while another set (i.e., VNTR-F and VNTR-R) amplifies the HVRii region. The reaction volume of PCR amplification was 25 μL, including 2.5 μL 10 × Trans TaqHiFi Buffer, 2 μL dNTPs (2.5 mmol·L^−1^), 1 μL of each primer (10μmol·L^−1^), 0.20 μL Trans Taq HiFi Polymerase (5 U·μL^−1^), 1 μL DNA template, and 17.7 μL ddH_2_O. The thermal reaction for the implication of HVRi region was started by an initial denaturation at 95°C for 2 min, followed by 27 cycles of denaturation at 95°C for 45 s, annealing at 64°C for 3 min, and elongation at 72°C for 3 min. The reaction was terminated by a final extension of 72°C for 10 min. The PCR amplification of HVRii region consisted of an initial denaturation at 95°C for 4 min, followed by 30 cycles of denaturation at 95°C for 45 s, annealing at 45°C for 45 s, and elongation at 72°C for 45 s, and ended with an elongation cycle at 72°C for 7 min. The HVRi and HVRii PCR products were separated by electrophoresis in 1.0 and 2.0 % (wt/vol) TBE buffer agarose gels at 110 V for 30 min and 100 V for 45 min, respectively. DNA markers MD5000 and MD500 (Transgen, Beijing, China) were used as molecular size standards for the HVRi and HVRii amplicons, respectively.

### Meteorological and Geographical Data of the Collection Sites

Monthly temperature data presented as an average over 15–30 years for each collection site was downloaded from the World Climate (http://www.worldclimate.com/) as described previously (Zhan and McDonald, 2011; Yang et al., 2016; Wu et al., 2020). Annual mean temperature at each site was estimated from the mean temperature over 12 months. Altitude, longitude, latitude, annual rainfall, and annual insolation duration data for each site were downloaded from the National Greenhouse Data System (http://data.sheshiyuanyi.com/WeatherData/). The annual rainfall and annual insolation duration data were presented as the accumulation of a year.

### Data Analysis

Mitochondrial haplotypes were assigned based on their PCR profiles amplified by the primers. Haplotype frequency was tabulated according to individual collections, and also their combination and heterogeneity within and among cropping regions were evaluated by a contingency χ^2^ test (Everitt, 1992). Haplotype diversity (HD) was estimated by Nei's index (Nei, 1978) for each individual collection. The overall population differentiation in mtDNA was estimated by *G*_*ST*_ (Nei, 1973) using mitochondrial haplotype frequency detected in each population, while the overall population differentiation in the nuclear genome was estimated by *F*_*ST*_ (Wright, 1943) using SSR data taken from previous publications (Qin et al., 2016; Yang et al., 2016). The standard deviation of *G*_*ST*_was generated by 100 resamples of original data with replacement, as described previously (Zhan et al., 2003), and used for a t-test to infer the evolutionary history of mtDNA (Yang et al., 2016). A higher *G*_*ST*_than *F*_*ST*_indicates that population genetic differentiation in the mitochondrial genome was caused by the natural selection for local adaptation while the lower *G*_*ST*_ than *F*_*ST*_indicates that mtDNA in *P. infestans* was generated by constraining selection for the same mitochondrial traits across regions. The hypothesis of neutral evolution in the mitochondria of *P. infestans will* not be rejected if *G*_*ST*_ does not differ from *F*_*ST*_.

The association of mitochondrial haplotype (frequency and diversity) with climatic (annual mean temperature, annual insolation duration, and annual rainfall) or geographic data (altitude; longitude, latitude, and longitude) in the collection sites were evaluated by linear and quadratic models. Climatic or geographic effect on haplotype frequency and diversity was evaluated by a multiple regression analysis using an analytical tool embedded in Microsoft Excel 2017.

The intrinsic growth rates of the isolates in a subset of populations were estimated by a logistic model (Aguayo et al., 2014) using the colony data taken from a previous publication (Yang et al., 2016). The colony sizes were measured during a10-day culture for each of the isolates at five experimental temperatures (13, 15, 19, 22, and 25 °C). The thermal reaction norm of the isolates was generated by fitting the *in-vitro* growth rates into a quadratic model. The expected maximum growth rates (MGR) of the isolates were calculated by taking the first derivative of the thermal reaction norm equation with the online derivative calculator (https://www.derivative-calculator.net/) and then solving the derivative after setting it to zero. MGRs were subjected to analysis of variance (ANOVA) by a General Linear Model (GLM) incorporated in SAS 9 and compared among haplotypes by the least-significant difference (LSD) test (Ott and Longnecker, 2015). In the GLM partition, “mitochondrial haplotype” was treated as a fixed variable, while isolates were treated as random variables. Haplotypes IR3 and IIR1 were absent in most of the isolated with the *in-vitro* growth rate data and were excluded from the GLM and LSD analysis.

## Results

### Mitochondrial Type Detection

**Figure 2:**
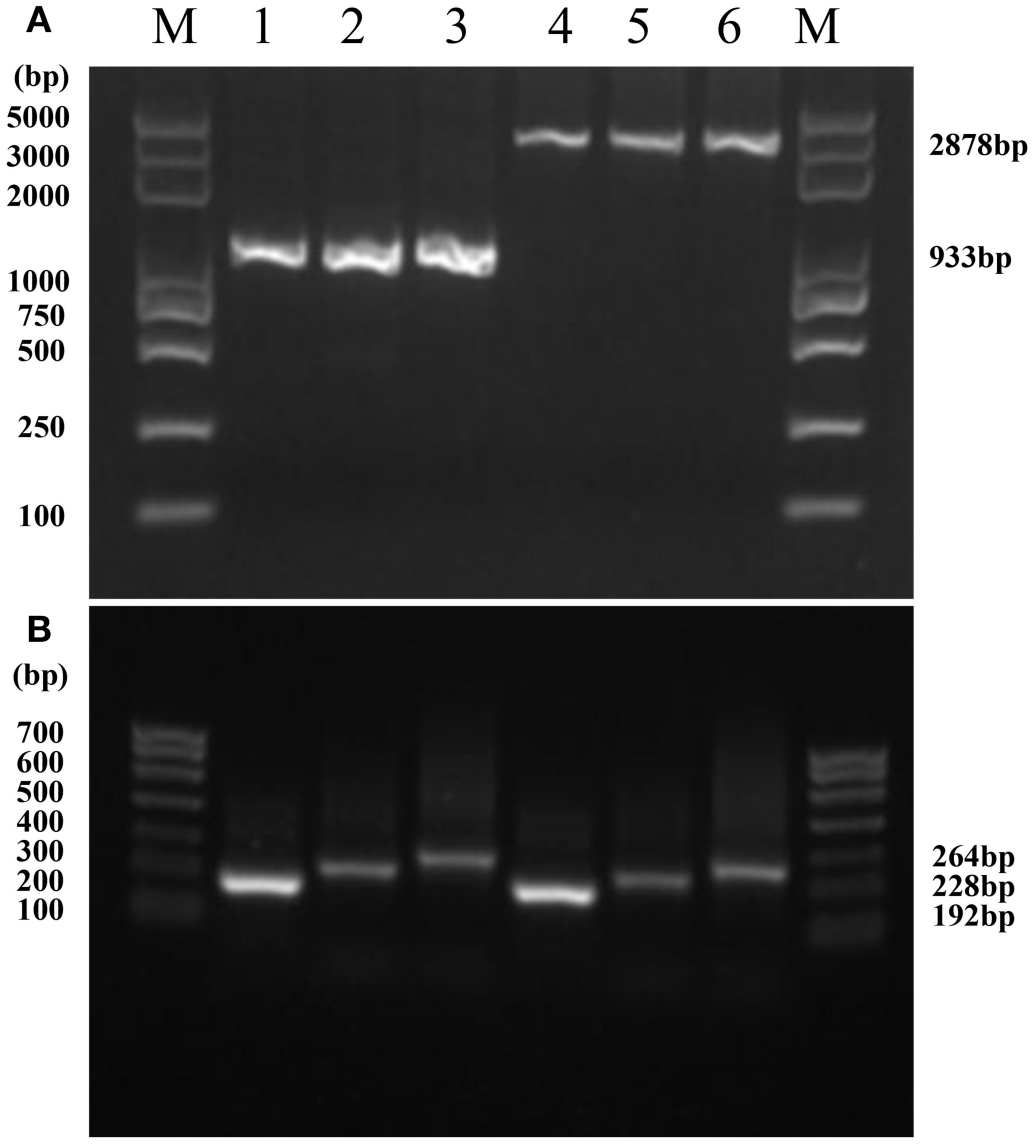
Amplicon profiles of *P. infestans* mitochondrial regions: **(A)** Band patterns of the mitochondrial region I (HVRI) amplified by the primers Insertion-F and Insertion-R. Lanes 1–3 represent mtDNA type I and lanes 4–6 represent mtDNA type II; M lanes are MD5000 DNA marker; **(B)**: Band patterns of the mitochondrial region II (HVRII) amplified by the primers VNTR-F and VNTR-R. Lanes 1 and 4 represent R1; lanes 2 and 3 represent R2; and lanes 5 and 6 represent R3. M lanes are MD700 DNA marker.

Only a single, clear, and reproducible band was generated by PCR amplification of the mtDNA of *P. infestans* from each isolate by each of the two pairs of primers (Figure 2). The HVRii primers generated two bands with a fragment size of 993 bp (type I, Figure 2A, lanes 1-3) and 2,878 bp (type II, Figure 2A, lanes 4-6) while HVRi primers yielded three bands with a size of 192 bp (Type R1, Figure 2B, lanes 1 and 4), 228 bp (Type R2, Figure 2B, lanes 2 and 3), and 264 bp (Type R3, Figure 2B, lanes 5 and 6), respectively. Therefore, the combination of the two primers can generate six mitochondrial types, and all of them were detected in the 405 isolates of *P. infestans* amplified in the study.

### Genetic Variation and Spatial Distribution of Mitochondrial DNA

The amplification assessment revealed a highly skewed band frequency in the *P. infestans* population from China (Table 2). Among the 405 isolates tested, 302 (74.57%) were classified as Type I and 103 (25.43%) as Type II. On the other hand, 174 (42.96%), 109 (26.91%), and 122 (30.10%) were designated as Type R1, R2, and R3, respectively. When the applications from the two sets of primers were considered together, IR1 was the most dominant mitochondrial haplotype, accounting for nearly half (42.72%) of the isolates assayed (Table 2), followed sequentially by IR2 (22.96%), IIR3 (21.23%), IR3 (8.89%), IIR2 (3.95%), and IIR1 (0.25%).

**Table 2:** Mitochondrial diversity and homogeneity test for haplotype frequency in the 15 *Phytophthora infestans* populations sampled from China.

**PCA**	**Regions**	**HD**	**Haplotype frequency**						**(χ^2^-test)**
			**IR1**	**IR2**	**IR3**	**IIR1**	**IIR2**	**IIR3**	
NSR	HHLJ	0.51	0.00	0.82	0.18	0.00	0.00	0	118.595 (12)*
	ARIM	0.33	0.06	0.19	0.67	0.00	0.06	0.03	
	TSGS	0.49	0.68	0.18	0.00	0.00	0.00	0.15	
	GYNX	0.41	0.71	0.29	0.00	0.00	0.00	0.00	
CDR	TXHN	0.43	0.24	0.00	0.00	0.05	0.00	0	42.335 (15)*
	ESHB	0.28	0.84	0.12	0.04	0.00	0.00	0.00	
	SZHB	0.66	0.52	0.10	0.05	0.00	0.14	0.19	
	WHHB	0.62	0.47	0.07	0.03	0.00	0.03	0.40	
SMR	ASGZ	0.45	0.70	0.23	0.00	0.00	0.00	0	16.67 (9)
	BJGZ	0.33	0.79	0.21	0.00	0.00	0.00	0.00	
	WZCQ	0.44	0.71	0.21	0.07	0.00	0.00	0.00	
	SZCQ	0.49	0.43	0.57	0.00	0.00	0.00	0.00	
SWR	FZFJ	0.58	0.03	0.44	0.00	0.00	0.06	0.	35.669 (8)*
	ZJGD	0.62	0.00	0.13	0.27	0.00	0.07	0.53	
	NNGX	0.62	0.15	0.10	0.00	0.00	0.18	0.56	

*PCA, Major potato cultivation areas; HD, Haplotype diversity*.

^*^: Significant at P < 0.0001.

The spatial distribution in the mitochondrial haplotypes was also highly skewed (Figure 1). The three dominant haplotypes, IR1, IR2, and IIR3 were underrepresented in the south, central, and south-western parts of China, representatively. IR3 was mainly restricted to the North part of the country, while IIR1 was only detected once at TXHN in CDR. Further analysis by homogeneity test showed a significant difference in haplotype frequency among field locations (χ^2^ = 477.07, *p* < 0.0001). Significant differences were also detected among field locations within three cropping regions (NSR, CDR, and SWR) (Table 2).

The haplotype diversity in the 15 locations ranged from 0.28 to 0.66 (Table 2) with an average of 0.48 (95% CI: ±0.06). Both the highest and the lowest diversity were found in the populations sampled from Hubei in CDR (SZHE and ESHB) on a regional scale. The pathogen population sampled from SWR had the highest haplotype diversity (0.61 ± 0.06) followed by those from CDR (0.50 ± 0.17) and NSR (0.44± 0.08). The population sampled from SMR had the lowest diversity (0.43 ± 0.07). However, the difference in haplotype diversity among the regions was insignificant by the LSD test (*p* = 0.1836). The population differentiation (*G*_*ST*_) in the mtDNA was 0.15, which was significantly higher than 0.08, the population differentiation (*F*_*ST*_) in SSR neutral markers by *t-*test (T = 5.67, *p* = 0.0024).

### Geographic Pattern of Spatial Distribution in Mitochondrial Haplotypes and Associations of the Distribution With Climatic Conditions

**Figure 3:**
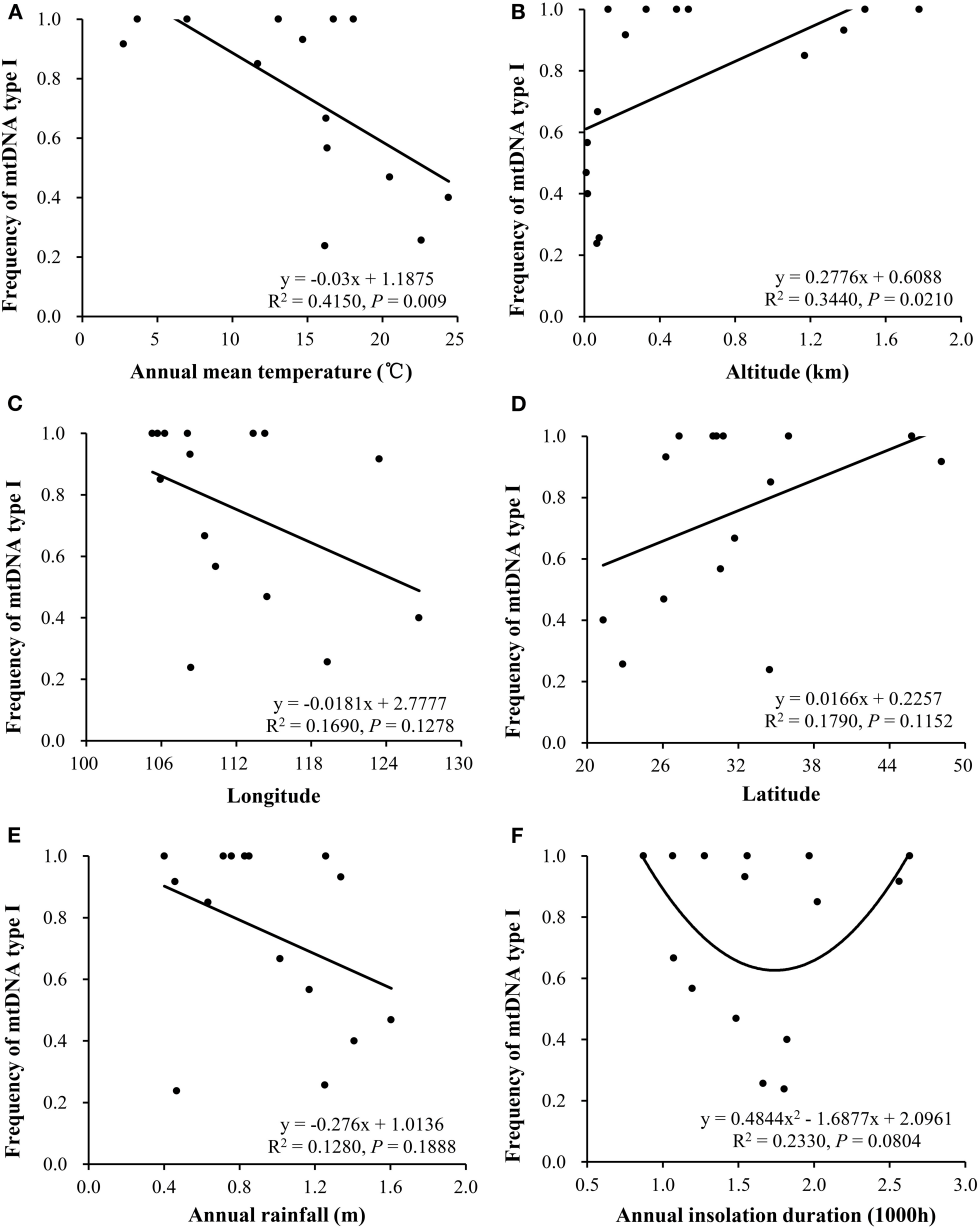
Associations of mitochondrial haplotype I with climatic conditions and tick in the *P. infestans* populations collected from China: **(A)** Annual means temperature, **(B)** altitude, **(C)** longitude, **(D)** latitude, **(E)** annual rainfall, and **(F)** insolation.

**Figure 4:**
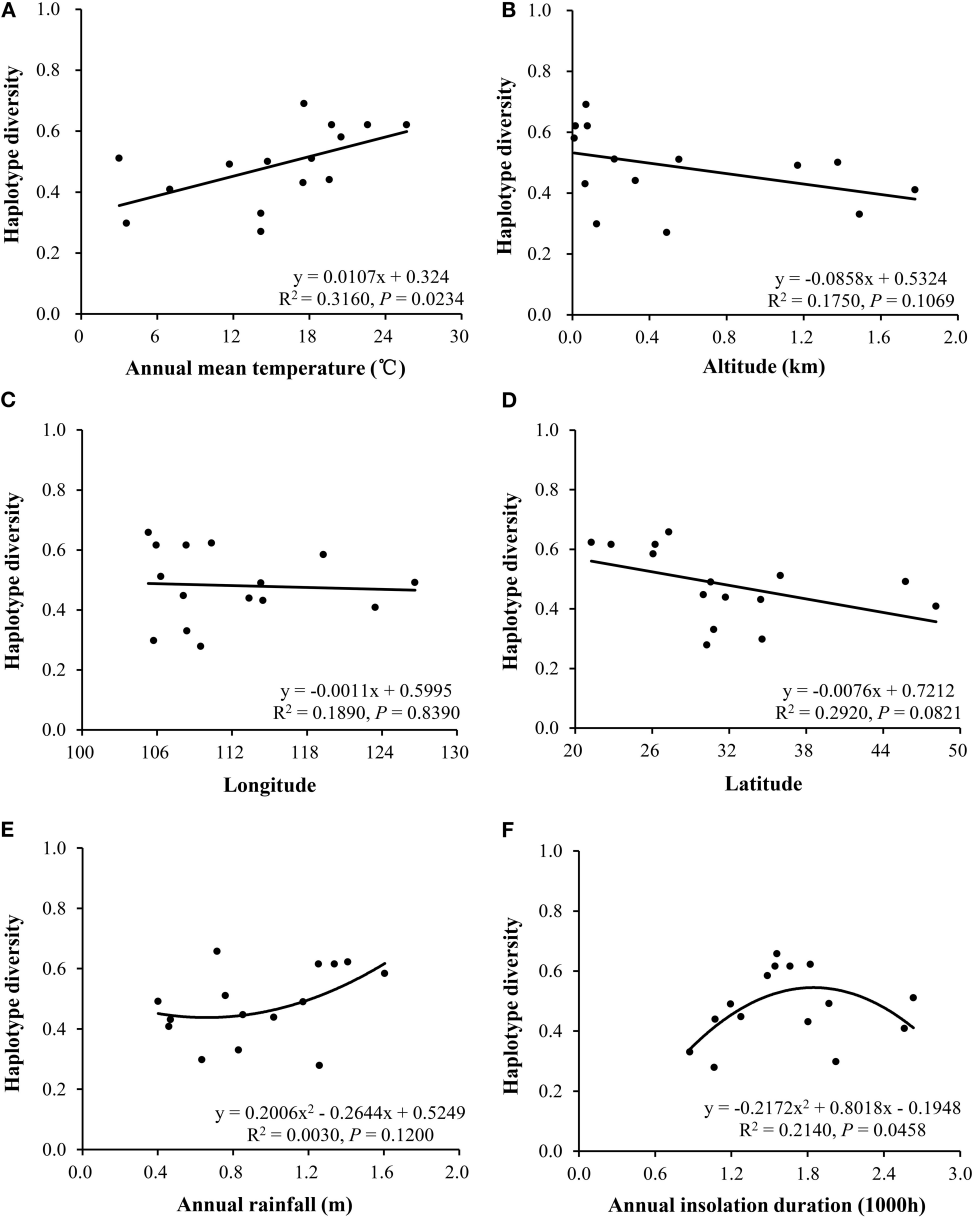
Correlation of annual mean temperature, altitude, latitude, and annual insolation duration of the collection sites with haplotype diversity in the *P. infestans* populations collected from China: **(A)** annual mean temperature, **(B)** altitude, **(C)** longitude, **(D)** latitude, **(E)** annual rainfall, and **(F)** insolation.

The annual mean temperature in the 15 collection sites was negatively correlated to the frequency of mitochondrial Type I (R^2^ = 0.4150, *p* = 0.0090, Figure 3A) but positively correlated to haplotype diversity (R^2^ = 0.3160, *p* = 0.0234, Figure 4A). Annual insolation duration in the collection sites was significantly and quadratically associated with haplotype diversity (R^2^ = 0.2140, *p* = 0.0458, Figure 4F) but only marginally associated with haplotype frequency (R^2^ = 0.2330, *p* = 0.0804, Figure 3F). On the other hand, altitude in the collection sites was significantly and linearly associated with haplotype frequency (R^2^ = 0.3440, *p* = 0.0210; Figure 3B) but only marginally associated with haplotype diversity (R^2^ = 0.1750, *p* = 0.1069, Figure 4B). Latitude was marginally associated with both haplotype frequency and diversity (Figures 3D, 4D). No associations were detected between other climatic conditions or geographic positions in the collection sites with haplotype (frequency and diversity). Multiple regression analysis also revealed that annual mean temperature and altitude in the collection sites contributed significantly to the spatial distribution of haplotype frequency and diversity (**Table 4**). On the other hand, annual insulation duration and latitude in the collection sites only significantly contributed to the spatial distribution of mitochondrial haplotype in term of frequency but not diversity.

### Differences in Temperature Response Among Mitochondrial Haplotypes

Exploratory analysis showed that MGR in the pathogen was normally distributed, therefore, original data were not transformed. Significant differences in the expected maximum intrinsic growth rate (MGR) were detected among the haplotypes by ANOVA (*p* = 0.001). The average MGR of Type I (0.5044 ± 0.0286) was significantly higher that of Type II (0.4774 ± 0.0248). Further analysis showed that MGR of IR1 and IR2 haplotypes was significantly higher than that of IIR3. No differences in MGR were detected among IR1, IR2, and IIR2, and IIR2 and IIR3 (Table 3). MGR of the haplotype was positively associated with its frequency (r = 0.9523, *p* = 0.0477).

**Table 3:** Least-significant difference (LSD) tests for difference in the estimated maximum growth rate (MGR) of different mitochondrial haplotypes.

**mtDNA**	**MGR**
IR1	0.508^A^
IR2	0.499^A^
IIR2	0.480^AB^
IIR3	0.477^B^

*Values followed by different letters in a column are significantly different from each other at P = 0.05*.

## Discussion

High population differentiation in the mitochondrial genome of *P infestans* was revealed by spatial analysis of haplotype frequency and diversity. Both deterministic and stochastic events can generate the observed spatial distribution. Natural selection for genetic variation ensuring a better chance of survival, reproduction, and transmission for a species in the specified ecological ecotypes such as community structure and climate conditions of a geographic location may lead to adaptive differentiation (Dumartinet et al., 2020). When gene flow is limited, stochastic changes of genetic variation in the mitochondrial genome domiciling in different geographic areas associated with genetic drift can lead to non-adaptive genetic differentiation (Zhan et al., 2003).

MtDNAs have historically been assumed to be selectively neutral (Galtier et al., 2009; Immonen et al., 2020). However, accumulating information shows that this molecule may be under strong selection for ecological adaptation (Zhan et al., 2004; Immonen et al., 2020), consistent with its involvement in the differentiation, information transmission and apoptosis of cells (Scheckhuber et al., 2007), regulation of body biomass (Illescas et al., 2021), response to environmental stresses such as pathogen resistance to host immunity (Bartelli et al., 2018) and pesticides, and we believe that natural selection for local adaptation also contributes to the spatial distribution of mtDNA observed in *P. infestans*. Our argument is supported by several lines of evidence as listed below.

As a proxy of ecotypes, many biotic and abiotic elements of an ecosystem, such as temperature, UV irradiation, oxygen level and species richness, density, and morphology are changed directionally in response to increase or decrease in altitude (Tanaka et al., 2019; Wang et al., 2020). For example, plants adapting to low altitudes tend to have long, thinner, and less hairy leaves than those adapting to high altitudes (Liu et al., 2020). The finding of linear association between haplotype diversity and altitude (Figure 4) indicates the spatial distribution of mitochondrial haplotypes is not random across ecosystems in China.

Differentiation caused by genetic drift is expected to have no impact on fitness (Orr, 2009). In this study, we find significant difference in intrinsic growth rate among the mitochondrial haplotypes (Table 3) and the difference can be successfully transferred to competitive ability as indicated by the positive association between haplotype frequency observed in nature and its intrinsic growth rate. Apparently, mitochondrial Type I is a more successful than Type II in *P. infestans*. It is the dominant haplotype, possibly attributed to its higher fitness, and the result is similar to other reports. For example, Type I dominated in the surveys conducted in India (Sharma et al., 2016), Turkey (Gunacti et al., 2019), as well as other parts of China (Yang et al., 2013) and adapts to wider ecological niches (Sharma et al., 2016).

A higher degree of population differentiation in mtDNA than neutral genome represented by SSR provides further evidence supporting an adaptive evolution (Wu et al., 2016; Yang et al., 2018; Shen et al., 2021; Waheed et al., 2021) in the *P. infestans* molecules. Gene flow is the main evolutionary mechanism determining the population differentiation of species (Gao et al., 2020). Low population differentiation (0.08) in neutral genome achieved by natural and/or human-mediated dispersal (Gao et al., 2020) is consistent with a high gene flow of the pathogen. The propagules of *P. infestans* can be transmitted to a long distance by air current (Firester et al., 2018), and intensive cultivation of its potato and tomato hosts year-round in the country (Guha Roy et al., 2021) creates a unique opportunity for transmission. In agriculture, anthropogenic activities such as commercial trading of plant materials by human activities are the main channels of plant pathogens dispersal (Meng et al., 2018). In China, a remarkably large amount of potato products is transported around the country annually either as seed tubers or foods (Gao et al., 2020).

Among the three most important climatic elements in agricultural ecosystems available in the historical weather records, the temperature is found to have a consistent association with the spatial distribution of mitochondrial haplotypes (Figures 3, 4, Table 4), suggesting that the thermal condition in the collection sites contributes greatly to the local adaptation of mtDNA in the pathogen, while other climatic elements might play no (e.g., rainfall) or less role (e.g., insolation). The mirrored associations of mitochondrial distribution with temperature and altitude may also reflect the importance of temperature in the adaptation because temperature always decreases as altitude increases. This argument is aligned with documented results in other species (Luo et al., 2013).

**Table 4:** Multiple regression analysis of the contribution of local climatic conditions or geographic positions to the mitochondrial genome in the *P. infestans* populations from China.

**Factor**	**Parameter**	**Type I frequency**		**Mitochondrial diversity**	
		**Coefficients**	***P*-values**	**Coefficients**	***P*-values**
Climates	AMT	0.0450 ± 0.0192	0.0003	0.0158 ± 0.0103	0.0058
	AID	0.0003 ± 0.0002	0.0077	0.0001 ± 0.0001	0.099
Coordinates	Altitude	0.0003 ± 0.0002	0.0107	0.0001 ± 0.0001	0.0483
	Latitude	0.0171 ± 0.0168	0.0470	0.0068 ± 0.0079	0.0841

*AMT, Annual mean temperature (°C); AID, Annual insolation duration (h)*.

Indeed, it has been shown that the spatial distribution of mtDNAs in many species in nature corresponds with their ability to adapt to thermal conditions. Over 10% of the mitochondrial haplotypes in fresh water gastropod sampled from a volcanic lake in northern Iceland were specified to a particular thermal habitat (Quintela et al., 2014) and mitochondrial haplotypes experienced stronger selection at high altitudes, where cold and hypoxic conditions are a major challenge for aerobic organisms (Camus et al., 2017). The ability of *Drosophila melanogaster* to tolerate thermal stresses in Australia was regulated by genomic variation of mitochondrial haplotypes, with the subtropical population displaying a greater resilience to heat stress but lower resilience to cold stress relative to temperate populations and vice versa (Camus et al., 2017). In *Saccharomyces cerevisiae*, mtDNA is an evolutionary hotspot for thermal adaptation where a large part of the genome is dedicated to develop heat and cold tolerance (Li et al., 2019). Similarly, low temperatures are selected for increasing metabolic efficiency in Atlantic salmon mtDNA (Consuegra et al., 2015).

## Conclusion

Global climates are undergoing an unprecedented change. It is projected that the air temperature of the planet would increase several degrees in the next 50 years, accompanied by more frequent heat and chilly waves (Masson-Delmotte et al., 2021). Given the critical role of temperature on the biological and evolutionary processes of species, these changes in global thermal profile greatly challenge ecological sustainability in terms of structure, function, or regulating disease epidemics. Consequently, understanding the mechanisms of thermal adaptation in species is necessary for the prediction and mitigation of the impacts. As the major manufacturer where cells carry out aerobic respiration, mitochondria are popularly known as the “powerhouse” of living organisms. They are involved in oxidative phosphorylation processes in which chemical energy from food and other resources are converted into adenosine triphosphate (ATP), the energy which is used for all metabolic processes (Sandor et al., 2018). The respiratory activities, and consequently the production of ATPs in cells, are environment-, particularly temperature-dependent. Low temperatures generally decrease the rate of cellular respiration due to the reduced kinetic energy required, leading to the differential suppression of relative genes and vice versa (Salomon and Buchholz, 2000). Therefore, it is expected that mtDNA plays a pivotal role in the thermal adaptation of species. However, research on the evolutionary mechanisms of thermal adaptation has mainly focused on the nuclear genome while the contribution of mtDNA to the adaptation is largely overlooked (Consuegra et al., 2015; Camus et al., 2017; Immonen et al., 2020). We believe that our research will draw more attention to narrow the gap.

## Data Availability Statement

The original contributions presented in the study are included in the article/supplementary material, further inquiries can be directed to the corresponding author.

## Author Contributions

L-LS and AW performed the experiments, analyzed data, and wrote the manuscript. Y-PW and ON collected pathogen isolates and genotyped pathogen isolates. Z-HW revised the manuscript. L-NY supervised the project and wrote the manuscript. JZ conceived, designed and supervised the experiments, analyzed the data, and wrote the manuscript. All authors reviewed the manuscript, contributed to the article, and approved the submitted version.

## Funding

This research was funded by the National Natural Science Foundation of China (31761143010, 31901861, and U1405213 grant to JZ and L-NY).

## Conflict of Interest

The authors declare that the research was conducted in the absence of any commercial or financial relationships that could be construed as a potential conflict of interest.

## Publisher's Note

All claims expressed in this article are solely those of the authors and do not necessarily represent those of their affiliated organizations, or those of the publisher, the editors and the reviewers. Any product that may be evaluated in this article, or claim that may be made by its manufacturer, is not guaranteed or endorsed by the publisher.
