# Corrigendum: Mitochondrial genome contributes to the thermal adaptation of the oomycete *Phytophthora infestans*

**DOI:** 10.3389/fmicb.2022.1002575

**Published:** 2022-08-11

**Authors:** Lin-Lin Shen, Abdul Waheed, Yan-Ping Wang, Oswald Nkurikiyimfura, Zong-Hua Wang, Li-Na Yang, Jiasui Zhan

**Affiliations:** ^1^Institute of Oceanography, Minjiang University, Fuzhou, China; ^2^Sichuan Provincial Key Laboratory for Development and Utilization of Characteristic Horticultural Biological Resources, Chengdu Normal University, Chengdu, China; ^3^Institute of Plant Virology, Fujian Agriculture and Forestry University, Fuzhou, China; ^4^Department of Forest Mycology and Plant Pathology, Swedish University of Agricultural Sciences, Uppsala, Sweden

**Keywords:** mitochondria, evolutionary ecology, population genetic, local adaptation, agricultural pathogen, climate change

In the published article, there was an error made during production in the manuscript text that should be written Table 4 instead of Table 3.

A correction has been made to **[Results]**, [**Geographic pattern of spatial distribution in mitochondrial haplotypes and associations of the distribution with climatic conditions]**, [Paragraph Number 5]. This sentence previously stated: “The annual mean temperature in the 15 collection sites was negatively correlated to the frequency of mitochondrial Type I (R^2^ = 0.4150, *p* = 0.0090, Figure 3A) but positively correlated to haplotype diversity (R^2^ = 0.3160, *p* = 0.0234, Figure 4A). Annual insolation duration in the collection sites was significantly and quadratically associated with haplotype diversity (R^2^ = 0.2140, *p* = 0.0458, Figure 4F) but only marginally associated with haplotype frequency (R^2^ = 0.2330, *p* = 0.0804, Figure 3F). On the other hand, altitude in the collection sites was significantly and linearly associated with haplotype frequency (R^2^ = 0.3440, *p* = 0.0210; Figure 3B) but only marginally associated with haplotype diversity (R^2^ = 0.1750, *p* = 0.1069, Figure 4B). Latitude was marginally associated with both haplotype frequency and diversity (Figures 3D, 4D). No associations were detected between other climatic conditions or geographic positions in the collection sites with the haplotype frequency and diversity. Multiple regression analysis also revealed that annual mean temperature and altitude in the collection sites contributed significantly to the spatial distribution of haplotype frequency and diversity (Table 3). On the other hand, annual insolation duration and latitude in the collection sites only significantly contributed to the spatial distribution of mitochondrial haplotype in terms of frequency but not diversity.”

The corrected sentence appears below:

“The annual mean temperature in the 15 collection sites was negatively correlated to the frequency of mitochondrial Type I (R^2^ = 0.4150, *p* = 0.0090, Figure 3A) but positively correlated to haplotype diversity (R^2^ = 0.3160, *p* = 0.0234, Figure 4A). Annual insolation duration in the collection sites was significantly and quadratically associated with haplotype diversity (R^2^ = 0.2140, *p* = 0.0458, Figure 4F) but only marginally associated with haplotype frequency (R^2^ = 0.2330, *p* = 0.0804, Figure 3F). On the other hand, altitude in the collection sites was significantly and linearly associated with haplotype frequency (R^2^ = 0.3440, *p* = 0.0210; Figure 3B) but only marginally associated with haplotype diversity (R^2^ = 0.1750, *p* = 0.1069, Figure 4B). Latitude was marginally associated with both haplotype frequency and diversity (Figures 3D, 4D). No associations were detected between other climatic conditions or geographic positions in the collection sites with haplotype (frequency and diversity). Multiple regression analysis also revealed that annual mean temperature and altitude in the collection sites contributed significantly to the spatial distribution of haplotype frequency and diversity (Table 4). On the other hand, annual insulation duration and latitude in the collection sites only significantly contributed to the spatial distribution of mitochondrial haplotype in term of frequency but not diversity.”

In the published article, there was an error made during production in the manuscript text that should be written Table 3 instead of Table 4.

A correction has been made to Discussion, Paragraph Number 4. This sentence previously stated: “Differentiation caused by genetic drift is expected to have no impact on fitness (Orr, 2009). In this study, we find a significant difference in intrinsic growth rate among the mitochondrial haplotypes (Table 4) and the difference can be successfully transferred to competitive ability as indicated by the positive association between haplotype frequency observed in nature and its intrinsic growth rate. Apparently, mitochondrial Type I is more successful than Type II in *P. infestans*. It is the dominant haplotype, possibly attributed to its higher fitness, and the result is similar to other reports. For example, Type I dominated in the surveys conducted in India (Sharma et al., 2016), Turkey (Gunacti et al., 2019), as well as other parts of China (Yang et al., 2013) and adapts to wider ecological niches (Sharma et al., 2016).”

The corrected sentence appears below:

“Differentiation caused by genetic drift is expected to have no impact on fitness (Orr, 2009). In this study, we find significant difference in intrinsic growth rate among the mitochondrial haplotypes (Table 3) and the difference can be successfully transferred to competitive ability as indicated by the positive association between haplotype frequency observed in nature and its intrinsic growth rate. Apparently, mitochondrial Type I is a more successful than Type II in *P. infestans*. It is the dominant haplotype, possibly attributed to its higher fitness, and the result is similar to other reports. For example, Type I dominated in the surveys conducted in India (Sharma et al., 2016), Turkey (Gunacti et al., 2019) as well as other parts of China (Yang et al., 2013) and adapts to wider ecological niches (Sharma et al., 2016).”

The authors apologize for this error and state that this does not change the scientific conclusions of the article in any way. The original article has been updated.

## Publisher's note

All claims expressed in this article are solely those of the authors and do not necessarily represent those of their affiliated organizations, or those of the publisher, the editors and the reviewers. Any product that may be evaluated in this article, or claim that may be made by its manufacturer, is not guaranteed or endorsed by the publisher.
